# Bacterial fitness shapes the population dynamics of antibiotic-resistant and -susceptible bacteria in a model of combined antibiotic and anti-virulence treatment

**DOI:** 10.1016/j.jtbi.2015.02.011

**Published:** 2015-05-07

**Authors:** Lucy Ternent, Rosemary J. Dyson, Anne-Marie Krachler, Sara Jabbari

**Affiliations:** a School of Mathematics, University of Birmingham, United Kingdom; b Institute of Microbiology and Infection, University of Birmingham, United Kingdom

**Keywords:** Antibiotic resistance, Anti-virulence drugs, Mathematical Modelling

## Abstract

Bacterial resistance to antibiotic treatment is a huge concern: introduction of any new antibiotic is shortly followed by the emergence of resistant bacterial isolates in the clinic. This issue is compounded by a severe lack of new antibiotics reaching the market. The significant rise in clinical resistance to antibiotics is especially problematic in nosocomial infections, where already vulnerable patients may fail to respond to treatment, causing even greater health concern. A recent focus has been on the development of anti-virulence drugs as a second line of defence in the treatment of antibiotic-resistant infections. This treatment, which weakens bacteria by reducing their virulence rather than killing them, should allow infections to be cleared through the body׳s natural defence mechanisms. In this way there should be little to no selective pressure exerted on the organism and, as such, a predominantly resistant population should be less likely to emerge. However, before the likelihood of resistance to these novel drugs emerging can be predicted, we must first establish whether such drugs can actually be effective. Many believe that anti-virulence drugs would not be powerful enough to clear existing infections, restricting their potential application to prophylaxis. We have developed a mathematical model that provides a theoretical framework to reveal the circumstances under which anti-virulence drugs may or may not be successful. We demonstrate that by harnessing and combining the advantages of antibiotics with those provided by anti-virulence drugs, given infection-specific parameters, it is possible to identify treatment strategies that would efficiently clear bacterial infections, while preventing the emergence of antibiotic-resistant subpopulations. Our findings strongly support the continuation of research into anti-virulence drugs and demonstrate that their applicability may reach beyond infection prevention.

## Highlights

• Combined antibiotic & anti-virulence drugs can clear antibiotic-resistant infections.
• Delay between delivery of drug types can render the treatment successful.
• Fitness cost associated with antibiotic-resistance shapes the optimal treatment.

## 1 Introduction

Bacterial resistance to antibiotic agents is an increasing problem in modern society. The introduction of every new class of antibiotic (from the original *β*-lactam, penicillin, to the more recent lipopeptides such as daptomycin) has been followed by the emergence of new strains of bacteria resistant to that class, many emerging in the clinic only a few years after the introduction of the drug (Butler and Buss, 2006; Clatworthy et al., 2007; Lewis, 2013). Given that the pace of antibiotic discovery has dramatically slowed down (most classes of antibiotic were discovered in the 1940s to the 1960s, the ‘Golden Era’ of antibiotics, with the past 40 years giving us only five significant new classes (Butler and Buss, 2006; Lewis, 2013) and pharmaceutical companies devoting less research into discovering new antibiotics (Projan and Shlaes, 2004; Mellbye and Schuster, 2011), this poses a huge problem. Development of novel treatments for bacterial infections is of utmost importance.

Traditional antibiotics are classed either as bactericidal or bacteriostatic, working to kill bacteria or inhibit bacterial growth respectively (Clatworthy et al., 2007; Mellbye and Schuster, 2011). While effective in eliminating susceptible bacterial infections, antimicrobials impose selective pressure on the bacteria, leading to the rise of resistant clones within the bacterial population. Resistance can be acquired either through spontaneous chromosomal mutation and then selection by antibiotic use, known as vertical evolution, or through acquiring genetic material from other resistant organisms, known as horizontal evolution (Tenover, 2006). Horizontal evolution occurs via mechanisms such as conjugation, transformation and transduction (Alanis, 2005) and can take place between strains of the same or different bacterial species. Horizontal evolution can lead to multidrug resistance and is a major concern in hospitals where resistant bacteria are able to remain viable despite antibiotic use and are the cause of many serious nosocomial infections in already vulnerable patients (Alanis, 2005; Tenover, 2006).

It has been suggested that the focus of new drug development should be on targeting virulence, the bacteria׳s ability to cause disease (Clatworthy et al., 2007), but this approach remains controversial both in terms of its potential efficacy and its ability to counter microbial resistance. Anti-virulence drugs would minimise any harm caused by bacteria while they remain in the host until they can be cleared by natural defences. This can occur either by being flushed out of the system or by being eradicated by the host׳s immune response. This *should* exert little to no selective pressure on the bacteria but this has not yet been proved *in vivo*. Anti-virulence drugs could target a range of mechanisms, including bacterial adhesion to host cells, toxin delivery or virulence gene regulation, all necessary for successful infection (Clatworthy et al., 2007; Mellbye and Schuster, 2011) and many of these are under investigation. In the vast majority of cases these approaches have proved to attenuate but not clear infection and often only when used in infection-prevention (as opposed to post-infection treatment), see for example Hentzer et al. (2003), Rasmussen et al. (2005), Wright et al. (2005), Jakobsen et al. (2012), Curtis et al. (2014), Hraiech et al. (2014), Sully et al. (2014) (quorum sensing and signalling inhibitors), Felise et al. (2008) (secretion inhibitor), Krachler et al. (2012) (adhesion inhibitor), Liu et al. (2008) (direct modulator of the bacteria׳s ability to suppress an immune response) and Hung et al. (2005) (colonisation inhibitor).

We adopt a modelling approach to investigate the viability of anti-virulence drugs *in silico*. Our results suggest that, in combination with antibiotics and under specific treatment strategies, anti-virulence drugs can be effective in clearing bacterial infections where antibiotic resistance is a concern. Optimal treatment strategies are likely to be specific to the patient, infection and bacterial strain and (rather than attempt to pin-point exact strategies) we use this theoretical framework as a “proof of concept”, exploiting parameter surveys to investigate a range of scenarios, highlighting the potential and the need for targeting infections with tailored treatments in the future. Given that every patient, infection and strain of bacteria are different, it is impossible to obtain a conclusive set of parameters which will suit all situations. We therefore exploit parameter surveys to ascertain under what conditions certain behaviour will occur.

This work lays the foundations for more detailed models incorporating mechanisms of specific types of anti-virulence drugs. This will facilitate testing of the likelihood that microbial resistance to these novel drugs could emerge. We address this briefly in Section 4 but leave this largely to further work due to the vast array of targets and resistance mechanisms that can be associated with anti-virulence drugs. Unless explicitly stated otherwise therefore, the description ‘resistant’ refers only to antibiotic-resistance in this work.

## 2 Materials and methods

### 2.1 Model formulation

There are two main approaches usually taken when modelling the emergence of antibiotic-resistant bacteria: within-host models, or within-hospital compartmental models. Models of hospital resistance usually follow a similar form to the classic “Susceptible–Infectious–Resistant” (SIR) models of epidemiology, looking specifically at how nosocomial infections will spread throughout the hospital, for example Austin and Anderson (1999), Lipsitch et al. (2000), and Webb et al. (2005). While such models are useful to develop strategies to prevent the spread of resistance, our focus is on treatment strategies to eliminate the emergence of an antibiotic-resistant subpopulation that has either arisen through random mutation and clonal expansion or through cross-contamination within a particular infection and under a specific treatment regimen. If the resistant bacteria within a single host can be eliminated then the spread of resistance throughout the hospital is a less pressing concern.

Existing mathematical models that focus on the within-host emergence of antibiotic-resistance examine how treatment strategies can both cause and be adapted to prevent the emergence of antibiotic resistance, for example Lipsitch and Levin (1997) and D׳Agata et al. (2008). Such models often neglect the effect of the host immune response, assuming it to be negligible or constant under the effect of the antibiotic. Since the efficacy of anti-virulence drugs will depend at least in part on the host׳s innate immunity, we include cell-mediated innate immune response in the model.

**Fig. 1:**
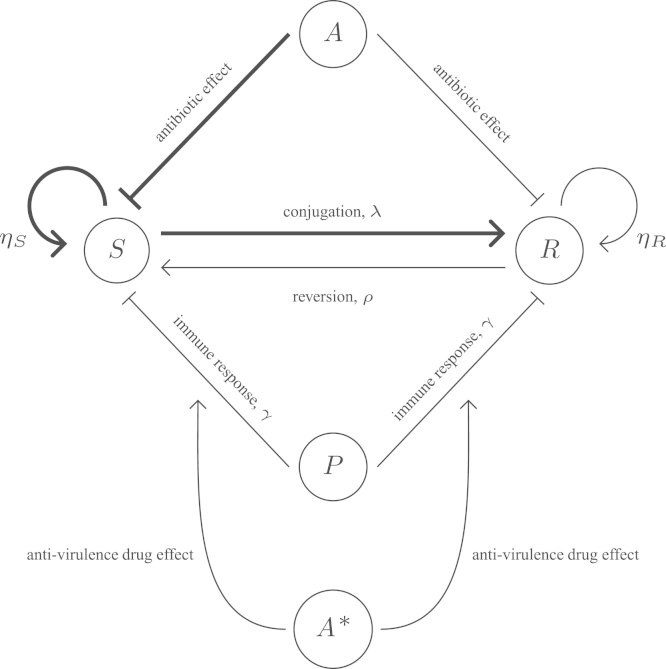
Schematic representation of the main interactions involved in an infection treated by antibiotics and anti-virulence drugs with *S* (antibiotic-susceptible bacteria), *R* (antibiotic-resistant bacteria), *P* (immune cells, e.g. phagocytes), *A* (antibiotic concentration) and *A*^⁎^ (anti-virulence drug concentration). Both the phagocytes and antibiotic inhibit bacterial growth, with the antibiotic having a greater effect on the susceptible bacteria than the resistant ones, assuming only partial resistance. The anti-virulence drug increases the effectiveness of the immune response in order to eliminate the bacteria more naturally, working on both susceptible and resistant bacteria in the same way.

The system consists of five interacting components: populations of antibiotic-susceptible bacteria (*S*), antibiotic-resistant bacteria (*R*) and immune cells e.g. phagocytes (*P*), and concentrations of antibiotic (*A*) and anti-virulence drug (*A*^⁎^). These interact as demonstrated in Fig. 1 and represent a generalised model of a local bacterial infection, such as a urinary tract or wound infection.

The growth of bacteria within a host is likely to saturate over time as a result of space and nutrient limitations. We therefore use a logistic growth term with baseline growth rate *η*_*i*_, (i=S,R) and a combined carrying capacity *K*, rather than simple exponential growth, to represent the bacterial dynamics. We include a removal rate, *ψ*, representing the body׳s endogenous, physical clearance mechanisms (this can be expected to vary depending upon infection type).

Due to the potential for multidrug resistance (Tenover, 2006), the primary cause for concern in hospitals is resistance due to horizontal evolution: acquiring new genetic material from other resistant organisms. Horizontal evolution involves the transfer of the resistance gene, normally found in sections of DNA known as transposons, from one plasmid to another. This takes place via one of three main mechanisms: conjugation, transformation or transduction (Alanis, 2005; Tenover, 2006). We focus on the most common of the three transmission mechanisms conjugation (Alanis, 2005), whereby one bacterium transfers plasmid containing the genes for resistance to an adjacent bacterium. Research suggests that these plasmid-bearing, and thus antibiotic-resistant, bacteria are subject to a fitness cost, *c*, lowering their growth rate (Levin et al., 1997; Austin and Anderson, 1999; Bergstrom et al., 2000; Lipsitch et al., 2000; Lipsitch, 2001), hence we choose the growth rate $\eta_{R}=(1-c)\eta_{S}$ where 0<c<1. Since this plasmid transfer occurs between adjacent bacteria, and we assume a well mixed population, we represent this interaction through mass action kinetics with a conjugation rate, *λ*, proportional to the levels of both antibiotic-susceptible and -resistant bacteria in the population (Bergstrom et al., 2000; Webb et al., 2005; Imran and Smith, 2006; D׳Agata et al., 2008). It has also been observed that it is possible to lose the plasmid carrying the resistance genes (Webb et al., 2005; Imran and Smith, 2006) and so this too is incorporated into the model via a constant reversion rate, *ρ*.

We assume that bacteria are consumed by phagocytes (immune cells) at rate *γ*. We note that the inclusion of a saturating effect on immune cell action, as seen in Malka et al. (2010), has no significant effect on the results in this study and we therefore omit it for simplicity. Rather than assuming a constant phagocyte level, as is often done, we incorporate a more realistic representation of cell-based, innate immune response into the model. Using a logistic style term, phagocytes are recruited to the site of infection at a rate proportional to the amount of bacteria present subject to an infection-site-specific, phagocytic carrying capacity, $P_{\max}$ (Smith et al., 2011). Phagocytes are lost through pathogen-induced apoptosis (at rate *δ*) and natural clearance (at rate $\delta_{P}$) (Zysk et al., 2001).

Antibiotic is either administered in one dose or assumed to be present at constant level (e.g. intravenous delivery) and rapidly absorbed to the site of infection (Austin and Anderson, 1999). Elimination of the antibiotic from the system, due to both degradation of the drug and clearance due to natural flow through the body, is assumed to be independent of the bacterial population and follows first order kinetics (Austin and Anderson, 1999; Imran and Smith, 2006). The effect of the antibiotic on each bacteria type is modelled using a saturating response, $E_{\max}^{i}A/(A_{50}^{i}+A)$, subject to a maximum killing rate $E_{\max}^{i}$ and the antibiotic concentration required for half maximum effect, *A*_50_^*i*^, for i=S,R (Lipsitch and Levin, 1997; Austin and Anderson, 1999; Nikolaou and Tam, 2006; Imran and Smith, 2007). Note that resistant bacteria are in reality often only partially resistant, thus in general we assume $E_{\max}^{R}\neq 0$ but $E_{\max}^{R}<E_{\max}^{S}$ (Lipsitch and Levin, 1997).

We assume that the anti-virulence drug increases the effectiveness of the host׳s immune response by weakening the bacteria׳s ability to counteract the host׳s immune mechanisms. The vast majority of bacterial virulence mechanisms interfere with the host immune response so this approach allows us to capture the action of a generic anti-virulence drug without needing to model specific mechanisms. Some anti-virulence compounds may directly inhibit the action of bacteria on immune cells (Liu et al., 2008, for example) while others have been shown to promote host clearance of the bacteria indirectly (e.g. quorum sensing inhibitors in Hentzer et al. (2003), Rasmussen et al. (2005), Jakobsen et al. (2012), Sully et al. (2014), or a secretion inhibitor in Felise et al. (2008)). Interference with adhesion mechanisms will also promote a host response since secretion systems responsible for production of virulence factors require host binding to function effectively (e.g. see Krachler et al., 2012). In order to model this generic anti-virulence drug mathematically we represent its effect as a saturating response, or Hill equation (Csajka and Verotta, 2006; Goutelle et al., 2008), with $\gamma_{\max}$ representing the maximum increased effect of the immune response and *γ*_50_, the anti-virulence drug concentration for half maximum effect. We assume that the anti-virulence drug has the same effect on both the antibiotic-resistant and susceptible bacteria.

Combining the above, we obtain the following model of bacterial population dynamics during antibiotic treatment:

(1) $$\frac{\mathrm{dA}}{\mathrm{dt}}=-\alpha A,$$

(2) $$\frac{{\mathrm{dA}}^{⁎}}{\mathrm{dt}}=-\kappa A^{⁎},$$

(3) $$\frac{\mathrm{dP}}{\mathrm{dt}}=\beta (S+R)(1-\frac{P}{P_{\max}})-\delta (S+R)P-\delta_{P}P,$$

(4) $$\frac{\mathrm{dS}}{\mathrm{dt}}=\{\begin{array}{cc} \eta_{S}S(1-\frac{S+R}{K})-\mu_{S}(A)S-(\gamma +\zeta (A^{⁎}))\mathrm{PS} & \\ \;-\lambda \mathrm{SR}+\rho R-\psi S, & \mathrm{if}\;S\geq 1,\\ -\psi S, & \mathrm{otherwise}, \end{array}$$

(5) $$\frac{\mathrm{dR}}{\mathrm{dt}}=\{\begin{array}{cc} (1-c)\eta_{S}R(1-\frac{S+R}{K})-\mu_{R}(A)R-(\gamma +\zeta (A^{⁎}))\mathrm{PR} & \\ \;+\lambda \mathrm{SR}-\rho R-\psi R, & \mathrm{if}\;R\geq 1,\\ -\psi R, & \mathrm{otherwise}, \end{array}$$

where the conditions on S,R<1 are included to ensure bacterial growth cannot resume in this regime. Here $\mu_{i}(A)=E_{\max}^{i}A/(A_{50}^{i}+A)$ is the effect of the antibiotic on susceptible and resistant bacteria, respectively, for i=S,R, and $\zeta (A^{⁎})=\gamma_{\max}A^{⁎}/(\gamma_{50}+A^{⁎})$ is the effect of the anti-virulence drug. Default parameter values and definitions are as given in Table 1, but a range of parameter values are explored throughout. Unless otherwise specified, we use the initial conditions:

(6) $$A(0)=4\;\mu g/\mathrm{ml},\;A^{⁎}(0)=4\;\mu g/\mathrm{ml},\;P(0)=0\;\mathrm{cells},S(0)=6000\;\mathrm{cells},\;R(0)=20\;\mathrm{cells},$$

representing clinically realistic dosages (for instance 4μg/ml is a MIC value for meropenem in *Pseudomonas aeruginosa* infections, see Liu et al., 2002) and the antibiotic-resistant subpopulation being in the minority and introduced via cross-contamination. We note that the initial conditions for numbers of bacteria are in line with the two studies from which we have principally sourced our parameter values – see Table 1, Handel et al. (2009) and Smith et al. (2011). Given that we are intending to provide a framework for future development of infection-specific treatment strategies and not proposing exact strategies here, these initial conditions are sufficient for our purposes.

The equations are solved numerically in Matlab using the solver *ode15s* for time-dependent solutions and multi-parameter steady-state analyses. The software XPPAUT is employed for single parameter steady-state surveys (where the S,R<1 conditions in (4) and (5) are removed for continuity). We consider three scenarios in our numerical solutions: an infection is treated by antibiotics alone, by an anti-virulence drug alone or by both drugs in combination. In each case we wish to see how effective the treatment strategies are in lowering bacterial load but crucially also in tackling the emergence of antibiotic-resistant bacteria during the infection.

## 3 Results

### 3.1 Antibiotic treatment (A(0)=4μg/ml, $A^{⁎}(0)=0\;\mu g/\mathrm{ml}$)

We consider a scenario where the bacterial population at the site of infection is composed predominantly of an antibiotic-susceptible subpopulation and a minor, resistant subpopulation which has arisen through cross-contamination of the site with a resistant population introduced from an environmental source (as is often the case during hospital-outbreaks) (Das et al., 2002; Fanci et al., 2009).

**Fig. 2:**
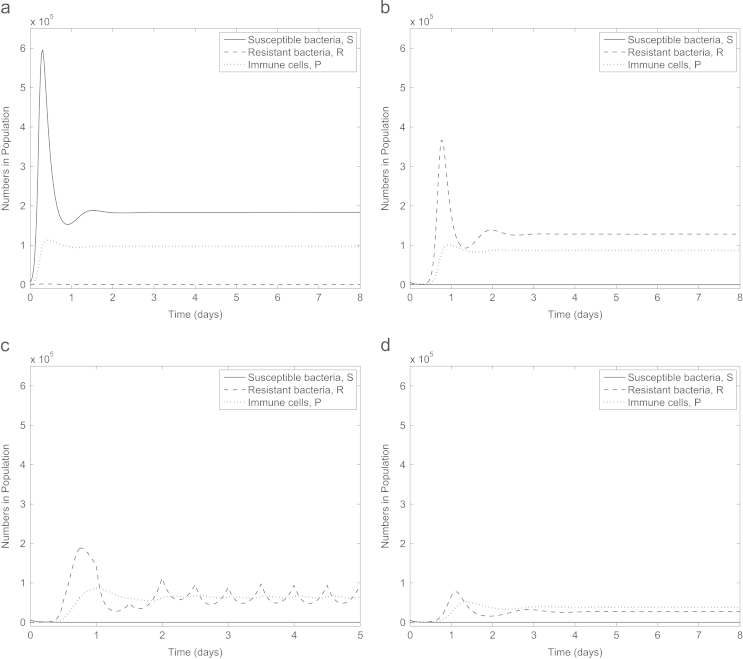
Numerical solutions to (1)–(5) with (a) no treatment ($A(0)=A^{⁎}(0)=0$), (b) one dose of antibiotic ($A(0)=4,A^{⁎}(0)=0$), (c) repeated dosing of antibiotic (*A* is reset to $4+A(\tau_{\mathrm{end}})$ at the start of each interval $\left[\tau \;\tau_{\mathrm{end}}\right)$ for τ=0.5p, p=1,2,3,…,9, $\tau_{\mathrm{end}}=\tau +0.5$ and $A^{⁎}(0)=0$) and (d) constant antibiotic level ($A(0)=4,A^{⁎}(0)=0,\alpha =0$). Our parameter choice reflects the situation where the immune system cannot clear the infection and a dominant population of antibiotic-resistant bacteria emerge if treated with antibiotics. Note that here and in subsequent figures, we have adjusted the minimum value on the *y*-axes to enable visualisation of any variables tending to zero.

Firstly we establish that our parameter set represents an infection where an initially small subpopulation of antibiotic-resistant bacteria emerges to become dominant when the infection is treated with antibiotics. In Fig. 2a (no treatment) susceptible bacteria remain dominant with the fitness costs associated with resistance meaning the antibiotic-resistant bacteria die out. One dose of antibiotic is introduced at time 0 in Fig. 2b, quickly killing the susceptible bacteria and providing an environment in which the resistant bacteria can flourish. Our parameter set therefore reflects a clinically relevant scenario.

Antibiotics can either be administered in doses (α>0) or continuously (α=0). Our model predicts that under antibiotic dosing all susceptible bacteria may be eradicated from the infection, however, as the antibiotic degrades out of the system the population of resistant bacteria increases exponentially (Fig. 2b and c). Upon a new dose of antibiotics being administered, the level of resistant bacteria drops slightly (due to the allowance of only partial resistance), subsequently quickly rising when the antibiotic degrades out of the system. Next, we consider constant antibiotic administration (e.g. via intravenous therapy, Fig. 2d). Susceptible bacteria are eliminated and the resistant bacteria reach a lower (but still high) steady state than in the absence of any drug. Thus the model reflects the type of infection in which we are most interested: one which cannot be cleared by either the immune system or antibiotics and in which antibiotic-resistance is a primary concern. For the remainder of this study we consider only constant antibiotic concentration for mathematical simplicity.

### 3.2 Antibiotic treatment: parameter analysis

**Fig. 3:**
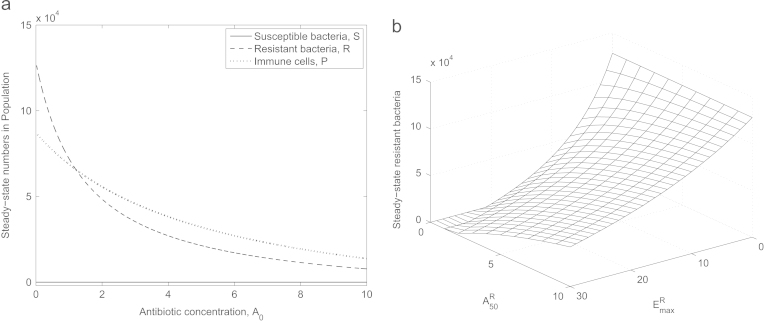
Steady-state values for varying (a) $A(0)=A_{0}$, (b) *A*_50_^*R*^ and $E_{\max}^{R}$ (the last two simultaneously). Note that here and henceforth we plot only stable steady-states to illustrate what is biologically feasible. In (a) we omit the behaviour for A(0)<0.01 where the switch between antibiotic-susceptible and -resistant bacteria dominating the population arises. Antibiotic-resistant bacteria will only be cleared by antibiotics (in combination with the immune system) if resistance is especially low ($A_{50}^{R}\to 0$ and $E_{\max}^{R}\to \infty$) or antibiotic concentration is very high.

Evidently, the amount of antibiotic administered will have a large effect on the final relative population levels of antibiotic-susceptible and -resistant bacteria (Fig. 3a). Though some resistant bacteria persist regardless of the dosage, as a result of the default parameter set representing only partial resistance, the bacterial load can be lowered by increasing the quantity of antibiotic administered (though A(0) extends beyond a clinically realistic range in Fig. 3a).

Partial resistance is governed by the two parameters $E_{\max}^{R}$ and *A*^*R*^_50_ (the antibiotic׳s maximum killing rate for resistant bacteria and the antibiotic concentration for half the maximum effect on resistant bacteria, respectively). The limits $E_{\max}^{R}\to 0$ or $A_{50}^{R}\to \infty$ yield complete resistance, and in Fig. 3b we see what happens when these parameters are varied in combination. The sensitivity of the bacteria to a specific antibiotic determines the bacterial load that persists in an infection treated by antibiotics: reducing the resistance level lowers this number.

Though the results so far are in no way surprising, they illustrate the power of adopting a modelling approach to predict the outcome of a particular infection: if the infecting strain can be isolated and the relevant parameters can be determined, it is possible to predict the number of bacteria remaining at an infection site and therefore identify an optimal treatment strategy.

**Fig. 4:**
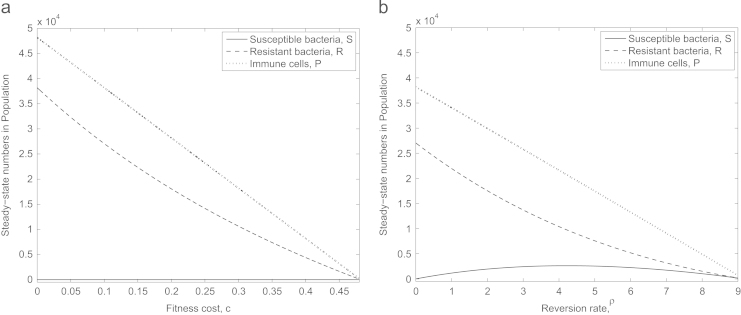
Steady-state values of *S*, *R* and *P* in response to varying (a) fitness cost, *c*, and (b) reversion rate, *ρ*. Sufficiently high *c* results in both subpopulations dying out (the susceptible bacteria as a result of the antibiotic and the resistant bacteria because the fitness cost renders them no longer viable) while there exists a range of *ρ* where *both* subpopulations can persist in the long-term in spite of treatment by antibiotic.

We recall that a fitness cost can be incurred as a consequence of the bacteria bearing the plasmid that confers antibiotic resistance. It has been shown that, depending on the plasmid in question, the fitness cost incurred can vary from 0.09 to 0.25 (Subbiah et al., 2011) as a proportion of the non-plasmid-bearing growth rate, and that over time this cost can reduce, sometimes eventually disappearing altogether (Dionisio et al., 2005). Thus, it is important to look at the effect of varying the cost of resistance on the whole infection (Fig. 4a). We observe that, as expected, a decrease in fitness cost increases the levels of resistant bacteria, and thus the level of immune cells (that are recruited in response to the presence of bacteria), at the infection site. As c→0, the plasmid is no longer exerting any significant metabolic cost on the bacteria and thus they are in a stronger position when faced with antibiotic pressure. Similarly, as *c* increases, we see the bacteria unable to compete against this antibiotic pressure and once c>0.48 (for our default parameter set), the benefit of resistance no longer outweighs the costs incurred and the antibiotic-resistant subpopulation dies out in the long term.

We also find that variations to *ρ*, the rate at which a plasmid-bearing bacteria loses its plasmid and reverts to being antibiotic-susceptible (Webb et al., 2005; Imran and Smith, 2006) can cause significant changes in the dynamics of the system (Fig. 4b). There exists a range of *ρ* where populations of both antibiotic-resistant and -susceptible bacteria are maintained in the population at steady state despite the presence of antibiotic: though the antibiotic kills the susceptible bacteria the resistant bacteria rejuvenate the susceptible population via plasmid loss. However, a sufficiently fast rate of plasmid loss results in both populations dying out: the resistant population all revert back to plasmid free susceptible bacteria leaving no plasmid-bearing bacteria to rejuvenate the population once the antibiotic has taken effect. In reality we expect this reversion rate to be very low so the situation whereby only resistant bacteria remain in the population is most likely (Sorensen et al., 2005).

### 3.3 Anti-virulence drugs (A(0)=0μg/ml, $A^{⁎}(0)=4\;\mu g/\mathrm{ml}$)

**Fig. 5:**
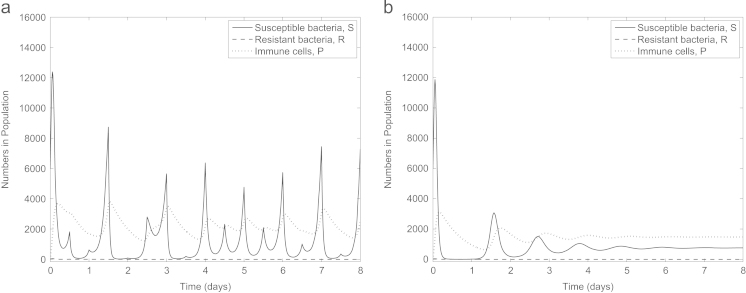
Numerical solution to (1)–(5) when the infection is treated only by anti-virulence drugs ($A(0)=0,A^{⁎}(0)=4$) (a) with doses every 0.5 days (κ=3.6) and (b) continuous treatment (κ=0). Though the antibiotic-resistant bacteria are quickly cleared from the infection, a subpopulation of antibiotic-susceptible bacteria persist because they grow at a faster rate than the antibiotic-resistant bacteria. From the scale of the axes, *S* appears to have vanished on some intervals, but S>1 holds for all time. The reduced number of immune cells present at the infection site as a result of the treatment lowering the total bacterial load leaves the immune response incapable of clearing the remaining susceptible bacteria. For the remainder of this study we consider only constantly administered anti-virulence drug for simplicity (κ=0).

We consider how effective anti-virulence drugs alone can be in treating a bacterial infection without antibiotics. Under our default parameter set (Fig. 5) the anti-virulence drug eliminates the antibiotic-resistant bacteria (this subpopulation is weaker due to the fitness cost imposed on them via the presence of the plasmid). However, as opposed to an infection treated only by antibiotics where antibiotic-resistant bacteria persisted, the anti-virulence drug fails to eliminate all the antibiotic-susceptible bacteria. Encouragingly though, the total number of bacteria remaining in the system is much lower than the number remaining after antibiotic treatment.

Despite the anti-virulence drug making the bacteria more vulnerable to the immune system there is still a limit to how effective the immune response can be and hence why some bacteria remain at the infection site. In modelling recruitment of the immune cells to the infection site, we imposed a recruitment rate, *β*, proportional to the number of bacteria present at the infection site, and a carrying capacity, $P_{\max}$. As such, the presence of immune cells is dependent on the presence of bacteria. As the drug starts to take effect the bacterial population diminishes, shortly followed by a decline in phagocyte population. Since this treatment is based on enabling the immune system to clear the infection, as the immune cell level decreases so does the efficacy of the drug; hence we see a possible reason for the persisting population of susceptible bacteria.

Thus although anti-virulence drugs are capable of dealing with the presence of antibiotic-resistant bacteria if they grow more slowly than antibiotic-susceptible bacteria, it may not be an effective treatment for the infection as a whole. In the following parameter analysis we consider whether anti-virulence drugs would ever be capable of clearing an infection or whether they might be more suitable for infection prevention.

### 3.4 Anti-virulence drugs: parameter analysis

A crucial aspect of an anti-virulence drug is its dependence on the immune system to be successful, and as such it may not be able to fully clear an infection under our default parameter set (Fig. 5). Both the natural removal rate, *ψ*, and the baseline immune response, *γ*, are not only very difficult to estimate, they are also patient- and site-specific. Along with inherent individual differences in patients, we also see variation within a single host depending on their current health. Since the main cause of concern for antibiotic resistance is in nosocomial infections (Alanis, 2005) patients may well be ill, immunosuppressed or debilitated before the onset of infection. This model is ideally posed to explore these parameters.

**Fig. 6:**
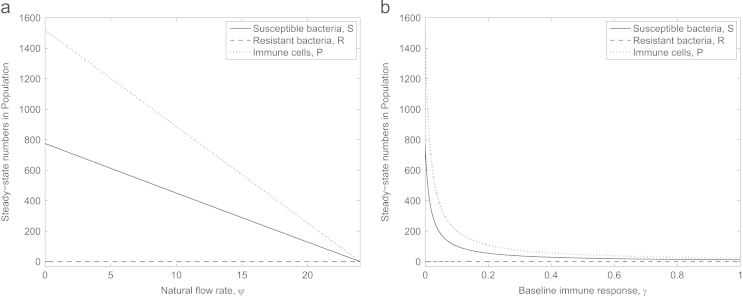
Steady-state values of bacteria and phagocyte levels under anti-virulence treatment when (a) natural clearance (*ψ*) and (b) baseline immune response (*γ*) are varied. The bacteria can be cleared from the infection but only when these host-specific parameters are sufficiently strong. We note that the critical values of *ψ* and *γ* above which all bacteria will be cleared are lower in the discontinuous model stated in (1)–(5) (around ψ≈12 and γ≈0.01) than in these figures due to the rule that *S* or *R* can only degrade when S<1 or R<1 in the time-dependent solutions (recall that this rule is removed to generate steady-state diagrams from continuous equations in XPPAUT).

Fig. 6 shows the effect of varying these host-specific parameters on the anti-virulence treatment. There exist circumstances when anti-virulence drugs can be completely effective, however these occur when either the baseline immune response or natural flow past the infection site is high. Thus it is possible that anti-virulence drugs may only be successful in either treating or preventing infection in an otherwise healthy individual. To test infection prevention in a model, a more sophisticated set of equations that captures a specific mechanism of action by an anti-virulence drug, as opposed to our deliberately general conceptual model, is required. This will be developed in future work.

In all cases examined here the population of antibiotic-resistant bacteria is eliminated, due to their reduced fitness making them more vulnerable to the treatment. Thus, for an existing infection requiring treatment, given that only susceptible bacteria remain, we next examine the option of combining treatment by anti-virulence drugs with conventional antibiotics.

### 3.5 Combination therapy: antibiotics and anti-virulence drugs (A(0)=4μg/ml, $A^{⁎}(0)=4\;\mu g/\mathrm{ml}$)

**Fig. 7:**
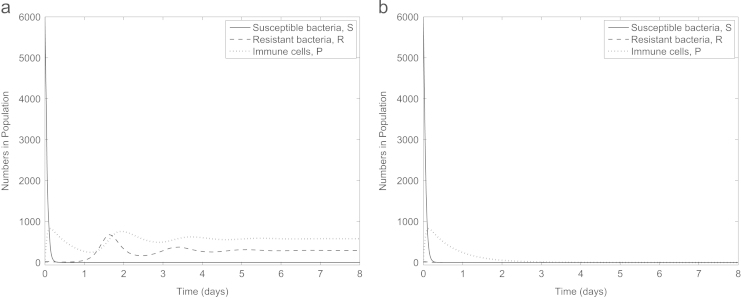
Simulation of combination therapy ($A(0)=A^{⁎}(0)=4,\alpha =\kappa =0$) with (a) *c*=0.1 and (b) *c*=0.27. The combined treatments are only successful if the fitness cost associated with antibiotic resistance is sufficiently high.

Our results so far suggest that anti-virulence drugs would rid an infection site of antibiotic-resistant but not antibiotic-susceptible bacteria, so it would be expected that the addition of antibiotics to this treatment would fully clear the infection. However, somewhat surprisingly, Fig. 7a indicates that this is not necessarily the case (even under the same default parameter set): though susceptible bacteria are cleared, a number of resistant bacteria persist if the fitness cost associated with antibiotic resistance is sufficiently low. This may be due to the dynamics of the immune cells: as the introduction of both drugs rapidly decreases the population of susceptible bacteria we also see a decline in the number of immune cells. Due to the dependence of the anti-virulence drug on the immune system, this decline may subsequently stunt the effectiveness of the drug, allowing the antibiotic-resistant population to build up to a level with which the immune system then cannot compete. In Fig. 7b, however, we see that raising the fitness cost to *c*=0.27 can result in both subpopulations being eliminated due to the additional weakness imposed on the antibiotic-resistant bacteria (we note that this is just outside the possible range for *c* suggested in Subbiah et al., 2011).

**Fig. 8:**
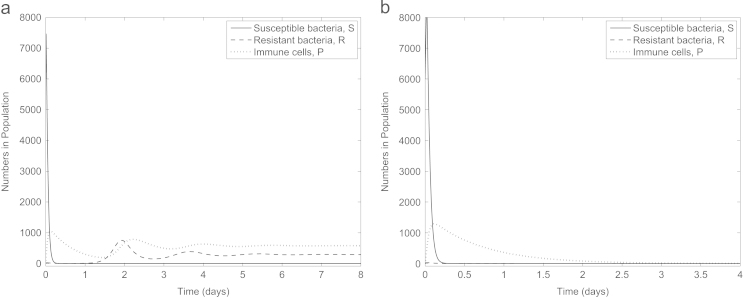
Predicted results of administering an anti-virulence drug to the infection site, with the addition of an antibiotic after (a) 0.01 days and (b) 0.02 days when *c*=0.1. If the delay between drug delivery is sufficient, the anti-virulence drug is able to eradicate the antibiotic-resistant bacteria, allowing the antibiotic to deal with the remaining susceptible bacteria.

**Fig. 9:**
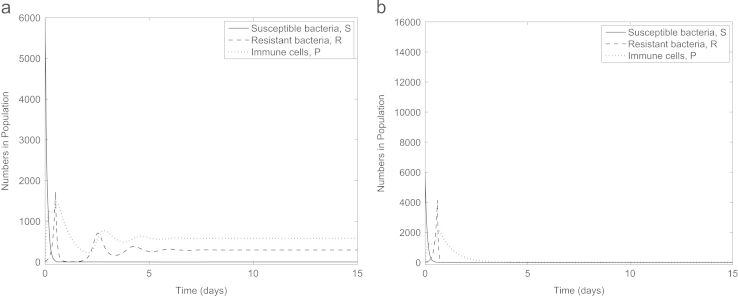
Predicted results of administering an antibiotic to the infection site, followed by an anti-virulence drug after (a) 0.5 days and (b) 0.6 days with *c*=0.1. The required delay between drugs for clearance of the infection is longer if the drugs are administered in this order than vice versa (compare with Fig. 8).

Excitingly, imposing a time delay on the administration of one of the drugs can allow combination therapy to be successful regardless of the fitness cost, see Fig. 8. When *c*=0.1, the infection can be fully cleared if the antibiotic is administered after the anti-virulence drugs, though the total bacterial load during this delay greatly increases. Reversing the treatment order is also successful, see Fig. 9. Here the delay is longer (and hence clearing the infection takes longer) but the increase in bacterial load before addition of the second drug is lower in this instance. Thus, we see that in combining two treatments, one which is more effective towards antibiotic-resistant bacteria and one which favours antibiotic-susceptible bacteria, we can effectively eliminate all bacteria provided a time delay is incorporated to enable both drugs to have full effect.

**Fig. 10:**
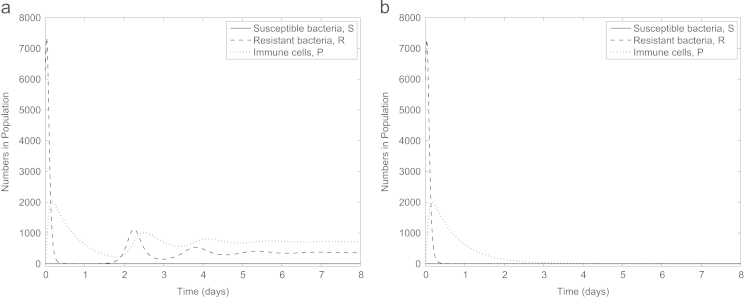
An infection consisting solely of (partially) antibiotic-resistant bacteria (S(0)=0,R(0)=6000) treated by both antibiotics and anti-virulence drugs simultaneously with (a) *c*=0.01 and (b) *c*=0.02. The combination therapy is capable of clearing the infection, but only if the fitness cost of antibiotic resistance is sufficiently high.

So far, all our simulations have focused on an infection where the antibiotic-resistant population are initially in the minority. In Fig. 10 we consider the effect of this combination therapy on an infection consisting entirely of antibiotic-resistant bacteria (S(0)=0,R(0)=6000). Interestingly, though neither drug in isolation will clear the infection (simulations not shown), the administration of both drugs with no time delay between them can clear the infection provided c>0.01 for our default parameter set. Thus combining antibiotics and anti-virulence drugs can even be successful in treating infections consisting solely of antibiotic-resistant bacteria initially (remembering that we are considering the bacteria to be partially resistant to the antibiotics and there is a low rate of reversion from resistant to susceptible). In addition, if we consider the scenario where antibiotic-resistance has occurred via spontaneous chromosomal mutation (hence λ=ρ=0) and no fitness cost is therefore incurred (*c*=0), delayed combination therapy again works, this time with delays of 0.03 days if the anti-virulence drug is administered first and 0.5 days if vice versa (default initial conditions used, data not shown), again underlining the need to fully understand the infection-type to design the treatment strategy correctly.

## 4 Discussion

Bacterial resistance to antibiotics is an increasing problem in today׳s society, more specifically in hospital settings where already vulnerable patients are exposed to strains of bacteria upon which conventional antibiotics have no effect. It has been widely suggested that in order to combat this emergence of resistance, research focus needs to shift from developing new antibiotics to novel treatment strategies that target bacterial virulence: an anti-virulence drug that promotes natural clearance through a weakening of their ability to counteract the immune response. This has given rise to much discussion of the potential success of such an approach with experimental investigations revealing, in general, attenuation but not clearance of infections. Would these drugs be better suited to infection prevention or can they be enhanced to treat existing infections? Since these drugs are still in the very early stages of development, we have developed a mathematical model to test their potential efficacy and treatment strategies *in silico*.

Our model suggests that, if used in isolation, an anti-virulence drug may not be capable of clearing an infection as it relies too heavily on the host immune system which was not strong enough to clear the infection itself in the first place (hence the need for treatment). The immune-related parameters are highly host-specific, though, and if taken to be representative of a healthy individual then the anti-virulence drug could well deal with an infection, suggesting that the drug could be useful for prophylaxis. However, since antibiotic resistance is mainly a problem in hospital settings, where patients are more vulnerable and are likely to have a weaker immune response, this dependence on the immune system is likely to be a limitation of the proposed treatment. Nevertheless, regardless of not being able to completely clear the bacterial infection in such immunocompromised patients, we do find some promising results. The persisting bacterial load is not only at a much lower level than the model predicted after antibiotic treatment (remembering that the simulated infection consists of a mixed population of antibiotic-susceptible and -resistant bacteria), but importantly the antibiotic-resistant bacteria were eliminated (due to the fitness cost associated with resistance) leaving only antibiotic-susceptible bacteria at the infection site. These results led us to investigate the potential of combination therapy. Treatment by both anti-virulence drugs and antibiotics could fully clear an infection if the fitness cost associated with antibiotic-resistance was sufficiently high. If this cost was lower, combination therapy could still be successful through the imposition of a time delay between treatment types, but the success of this approach was sensitive to the choice of delay between the drug types. This ‘delay’ result is not intuitive and highlights the benefits of adopting a modelling approach.

While underpinning the potential of anti-virulence drugs in a theoretical framework, this study also highlights the benefits of better understanding patient- and site-specific parameters pertinent to an infection in order to use a mathematical model to predict required treatment strategies. Though some (but not all) of the parameter surveys presented in this study are fairly intuitive, they illustrate the usefulness of mathematical models in this context. Treatment strategies are likely to be dependent on specific parameters within an infection, for example the level of resistance the infecting strain displays to a particular antibiotic, the likely bacterial load at an infection site or the time taken for treatment to reach the infection site. Thus, mathematical modelling will prove an extremely useful tool to provide tailored treatments that will optimise our use of drugs (unlike what has been seen with the over- and often mis-use of antibiotics).

The model can be extended to represent a specific anti-virulence drug. For example, anti-adhesive drugs that prevent bacteria binding to host cells should increase the natural clearance rate and this can be accounted for in extensions to the model. Such efforts will greatly enhance the predictive powers of this type of model and contribute to the future design of effective treatment regimen for bacterial infections. Crucially, these extensions to the model will also facilitate predictions on the likelihood that bacteria develop resistance to anti-virulence drugs in general and on whether the specific target of the drug will impact upon this, as argued in Allen et al. (2014). Our preliminary modelling investigations suggest that anti-virulence drug-resistance could emerge during an infection. However, for anti-virulence drug-resistance to emerge, the bacteria need to be more resilient to the drug than they do against antibiotics for antibiotic-resistance to emerge. We stress, though, that these are very early findings: the breadth of anti-virulence targets and resistance mechanisms makes this a task beyond the scope of the current study.

## 5 Conclusions

Antibiotic resistance in bacteria has long been recognised as a problem for effective treatment of infections, yet it is only more recently that the urgent need for antibiotic alternatives has become widely accepted. We have presented here a model of a generalised treatment strategy for changing the target of the drug to promote natural clearance. Our results support the continuation of research into anti-virulence drugs both in the context of treating infections and in aiding with the elimination of the spread of antibiotic-resistant bacteria from an infection and through hospital settings.

Throughout our analysis of these models we have highlighted some important issues that need to be considered when designing treatment strategies such as the importance of tailoring treatment to specific conditions of the host and how the success of combination therapy (use of more than one drug type) depends critically on the dosing schedule. Most importantly, however, we have shown conditions under which treatment strategies will be successful (some of which are non-intuitive) and hence provided a proof of concept for the potential such treatments should have to help eliminate the problem of antibiotic resistance.

## Figures and Tables

**Table 1:** Parameter notation with descriptions, estimated values and units. Immune-related parameter values are taken from Smith et al. (2011) and bacteria- and antibiotic-associated values from Handel et al. (2009). Where no source is given estimates have been made from what we believe to be realistic parameter ranges, in keeping with the other parameters. In particular *β* is chosen so that the immune system alone cannot clear the infection, the anti-virulence drug is assumed to have equivalent efficiency to the antibiotic, and *λ* and *ρ* are chosen so that an antibiotic-resistant strain can emerge to become dominant during infection under antibiotic selection. While we do not claim that these parameters represent one particular infection-type, they should fall within a realistic range, enabling us to investigate a spread of scenarios through variations to these values in the parameter surveys provided throughout the study.

**Parameter**	**Description**	**Units**	**Estimated value**	**Source**
*α*	Elimination rate of antibiotic under distinct doses	days^−1^	3.6	Handel et al. (2009)
	Elimination rate of antibiotic under intravenous therapy	days^−1^	0	–
*κ*	Elimination rate of anti-virulence drug	days^−1^	3.6	–
	Elimination rate of anti-virulence drug under intravenous therapy	days^−1^	0	–
*β*	Recruitment rate of phagocytes	days^−1^	3	–
*c*	Fitness cost of resistance	dimensionless	0.1	–
$\delta_{P}$	Clearance rate of phagocytes	days^−1^	1.512	Smith et al. (2011)
*δ*	Bacterial induced death of phagocytes	cells^−1^ days^−1^	$6\times 10^{-6}$	Smith et al. (2011)
*A*^*S*^_50_	Antibiotic concentration for half maximum effect on susceptible bacteria	μg/ml	0.25	Handel et al. (2009)
*A*^*R*^_50_	Antibiotic concentration for half maximum effect on resistant bacteria	μg/ml	5	Handel et al. (2009)
$E_{\max}^{S}$	Maximum killing rate of susceptible bacteria	days^−1^	36	Handel et al. (2009)
$E_{\max}^{R}$	Maximum killing rate of resistant bacteria	days^−1^	26.4	Handel et al. (2009)
*γ*_50_	Anti-virulence drug concentration for half maximum effect	μg/ml	5	–
$\gamma_{\max}$	Maximum increased effectiveness of immune response	cells^−1^ days^−1^	0.035	–
*K*	Carrying capacity of bacteria	cells	10^9^	Handel et al. (2009)
$\eta_{S}$	Growth rate of susceptible bacteria	days^−1^	24	Handel et al. (2009)
$P_{\max}$	Maximum number of phagocytes	cells	$1.8\times 10^{5}$	Smith et al. (2011)
*γ*	Bacterial clearance by phagocytes	cells^−1^ days^−1^	$2.4\times 10^{-4}$	Smith et al. (2011)
*λ*	Conjugation rate constant	days^−1^	10^−5^	–
*ρ*	Reversion rate constant	days^−1^	10^−6^	–
*ψ*	Removal rate	days^−1^	0.7	–
